# A Hybrid Wavelet-Based Deep Learning Model for Accurate Prediction of Daily Surface PM_2.5_ Concentrations in Guangzhou City

**DOI:** 10.3390/toxics13040254

**Published:** 2025-03-28

**Authors:** Zhenfang He, Qingchun Guo, Zhaosheng Wang, Xinzhou Li

**Affiliations:** 1 School of Geography and Environment, Liaocheng University, Liaocheng 252000, China; guoqingchun@lcu.edu.cn; 2 Institute of Huanghe Studies, Liaocheng University, Liaocheng 252000, China; 3 State Key Laboratory of Loess and Quaternary Geology, Institute of Earth Environment, Chinese Academy of Sciences, Xi’an 710061, China; lixz@ieecas.cn; 4 National Ecosystem Science Data Center, Key Laboratory of Ecosystem Network Observation and Modeling, Institute of Geographic Sciences and Natural Resources Research, Chinese Academy of Sciences, Beijing 100101, China; wangzs@igsnrr.ac.cn

**Keywords:** deep learning, ANN, LSTM, GRU, CNN, BiLSTM, BiGRU, PM_2.5_, wavelet

## Abstract

Surface air pollution affects ecosystems and people’s health. However, traditional models have low prediction accuracy. Therefore, a hybrid model for accurately predicting daily surface PM_2.5_ concentrations was integrated with wavelet (W), convolutional neural network (CNN), bidirectional long short-term memory (BiLSTM), and bidirectional gated recurrent unit (BiGRU). The data for meteorological factors and air pollutants in Guangzhou City from 2014 to 2020 were utilized as inputs to the models. The W-CNN-BiGRU-BiLSTM hybrid model demonstrated strong performance during the predicting phase, achieving an R (correlation coefficient) of 0.9952, a root mean square error (RMSE) of 1.4935 μg/m^3^, a mean absolute error (MAE) of 1.2091 μg/m^3^, and a mean absolute percentage error (MAPE) of 7.3782%. Correspondingly, the accurate prediction of surface PM_2.5_ concentrations is beneficial for air pollution control and urban planning.

## 1. Introduction

Surface air pollution affects the ecological environment, plant growth, climate change, food production, sustainable social development, and human health [1,2,3,4,5,6,7,8,9]. Nine out of ten people worldwide breathe polluted air, which causes probably seven million premature deaths each year [10]. Exposure to ambient fine particulate matter (PM_2.5_) causes more than four million premature deaths worldwide each year [11]. Therefore, the accurate prediction of surface PM_2.5_ is of great significance for human health, air quality management, urban planning, and government decision-making.

The highly nonlinear and complex relationship between meteorological variables and surface PM_2.5_ concentrations is often encountered, which makes it impossible to map using conventional statistical regression models and nonlinear statistical models, including linear regression, generalized linear regression, land-use regression, geographically weighted regression, autoregressive moving averages, and several nonlinear statistical regression models [12,13,14]. With the development of artificial intelligence (AI), it has been widely applied in many fields [15,16,17,18,19]. Machine learning (ML)-based models like artificial neural networks (ANNs) that have become increasingly fashionable in air pollution prediction provide an attractive alternative [20,21,22]. Wavelet-ANN (WANN) has the characteristics of nonlinearity, adaptability, and self-organization. It performs better than ANN in predicting PM_2.5_ time series, but WNN still has the problems of slow convergence speed and low prediction accuracy [23,24].

In recent years, deep learning (DL) techniques have more parameters and deeper structures, which can extract low-level features from original data and improve forecast performance [25,26,27]. Thanks to multi-layer learning, DL can also exactly approximate highly complex nonlinear relations and demonstrate advantages over traditional ML [28,29]. More and more environment researchers have applied deep learning models to environment research [16,30,31,32,33]. The DL algorithm has acquired good outcomes in the analysis and forecast of PM_2.5_, but there are still some challenges that need further in-depth research. CNN can extract features from time-series data, but it cannot uncover the problem of long dependencies in time-series data, and a single CNN model cannot fully capture the time-series information of historical data [34,35]. Therefore, RNN with strong modeling ability for time series can be introduced. The RNN has significantly improved the forecast accuracy of PM_2.5_ concentrations in the Seoul metropolitan area [36]. Specifically, more complex model units have been developed in RNN using gating mechanisms. GRU, as a variant of RNNs, can not only process time-series information but also effectively solve the problem of model gradient vanishing. However, GRU only focuses on the forward sequence information of the time series and does not consider the correlation of the reverse sequence [37]. However, Bidirectional GRU (BiGRU) can be used to fully capture the long-term dependencies present in the time series [38]. The internal structure of LSTM allows the network to selectively retain or forget information, which helps to handle long-term dependencies in temporal data [39]. Compared with RNN and multivariate linear regression (MLR), the LSTM model has a higher performance in predicting particulate matter (PM) in South Korea [40,41]. However, for longer time series, LSTM may forget earlier information and, therefore, cannot learn all the content of the data [42,43]. Bidirectional LSTM (BiLSTM) can consider the global information of the data and avoid forgetting earlier content due to too long temporal data. Meanwhile, BiLe BiLSTM model results show good performance in predicting time series of daily PM_2.5_ concentrations [44]. When considering robustness, any single artificial STM can effectively learn time-series data and solve the problem of long dependencies that CNN cannot handle [45]. The intelligence model has many limitations [46].

However, the combination model can compensate for the shortcomings of various models, capture the characteristics of daily PM_2.5_ concentrations, and complete good forecast performance. Consequently, combination strategies are more suitable for forecasting air pollutants. A handful of studies have developed CNN-ANN, CNN-RNN, CNN-GRU, CNN-LSTM, CNN-BiGRU, and CNN-BiLSTM models [47,48,49,50,51]. The hybrid CNN-GRU method can learn the variability and complexity of time series, aiming to predict the concentration of PM_2.5_. The 3D CNN-GRU model exhibited better performance than methods such as ARIMA, ANN, support vector regression machine (SVR), GRU, and LSTM [49]. The hybrid CNN-LSTM model outperformed the other models in predicting PM_2.5_ concentration [52]. The hybrid CNN-GRU-LSTM method remarkably improves the performances of CNN, LSTM, and GRU methods in forecasting PM_2.5_ concentrations in Dezhou City [53]. The performance of the 1D-CNN BiLSTM model for PM_2.5_ forecasting is better than that of the BiLSTM model. One reason may be that 1D-CNN BiLSTM can use three different pooling sizes to obtain multi-scale temporal information [54]. The proposed CLSTM-BiGRU model integrates a CNN, an LSTM, and a BiGRU network. The CLSTM-BiGRU model demonstrates its superiority in air pollutant prediction and outperforms the baseline (CLSTM, DTR, BiGRU) methods [55]. The MTCAN method combines the fast feature extraction ability of CNN and the temporal modeling features of RNN, improving PM_2.5_ prediction accuracy. Moreover, the MTCAN method performance is compared with statistical methods such as SARIMA, ML methods such as SVR and ANN, and deep learning methods such as LSTM, CNN, BiLTM, GRU, and BiGRU. Specifically, the MTCAN method has the best performance among the benchmark methods [56]. A Learning Rate Schedule (LRS) is introduced in deep learning models. Compared with the RMSE of the other methods, LRS-BiLSTM-CNN has the highest prediction performance in forecasting PM_2.5_ concentrations [57]. The findings revealed that the proposed model provided more accurate predictions. The GCN-LSTM-ResNet model significantly improved hourly PM_2.5_ concentration prediction performance. It reduced MAE by about 10.6–20.0% and reduced RMSE by about 13.2–17.1% [58]. The SA–EMD–LSTM method for long-term PM_2.5_ prediction is more accurate compared to other benchmark models [59]. CSBO-VMD-QRGRU-MGO-LSSVM is proposed to predict PM_2.5_ concentration, and the model has the greatest prediction precision [60]. Wavelet transform is suitable for feature extraction and the noise removal of time-series signals and can obtain the high-frequency components of input time-series signals, making it very suitable for handling scenarios with nonlinear PM_2.5_ fluctuations. Wavelet-based deep learning models can effectively overcome the problem of insufficient data over the training period and improve the accuracy of model forecasts [61]. To determine the optimal wavelet’s layers in forecasting PM_10_, a coupled WT-LSTM-SAE method is developed. The results show that the coupled method outperforms BiLSTM and LSTM [62]. The above results indicate that the hybrid models are more suitable for the combination of wavelet transform than the single models in predicting PM_2.5_ concentrations.

However, these studies still have some shortcomings: (1) PM_2.5_ concentration is prone to change over time and seasons, making it difficult for unoptimized prediction models and single prediction models to achieve high prediction accuracy. (2) PM_2.5_ concentration is easily affected by related air pollutants and meteorological factors, which can interfere with prediction accuracy. Not considering these factors will result in poor prediction accuracy. (3) The selection of prediction methods is relatively simple, and most prediction models continue to use simple prediction methods, so there may be some limitations in the accuracy and stability of predictions. (4) The wavelet function affects the prediction results, but its selection is usually subjective, which leads to poor decomposition performance.

Based on the above analysis, there is an urgent need to develop a high-precision hybrid optimization prediction model. Here, our study aims to predict air pollution more accurately using wavelet-based deep learning models. We develop a multi-factor PM_2.5_ optimization prediction model based on wavelet (W), CNN, BiGRU, and BiLSTM, named W-CNN-BiGRU-BiLSTM. Meanwhile, different factors are considered in the models, such as meteorological variables and air pollutants. The input predictors (parameters) are selected by R. By considering time dependence and frequency correlation, wavelet transform is applied to decompose the meteorological data and the air pollutant data into sub-level sequences. The selections of wavelet functions and parameters are accomplished through an optimization algorithm. Different machine learning models and wavelet-based hybrid models are used to forecast daily PM_2.5_ concentrations in Guangzhou City. Among all the models under investigation, the W-CNN-BiGRU-BiLSTM model exhibits the best performance. In summary, this study constructed a wavelet-based deep learning prediction model, which can provide efficient and simplified models for air pollution prediction and help further explore the application of the networks in other fields.

## 2. Data and Methods

### 2.1. Data

**Figure 1:**
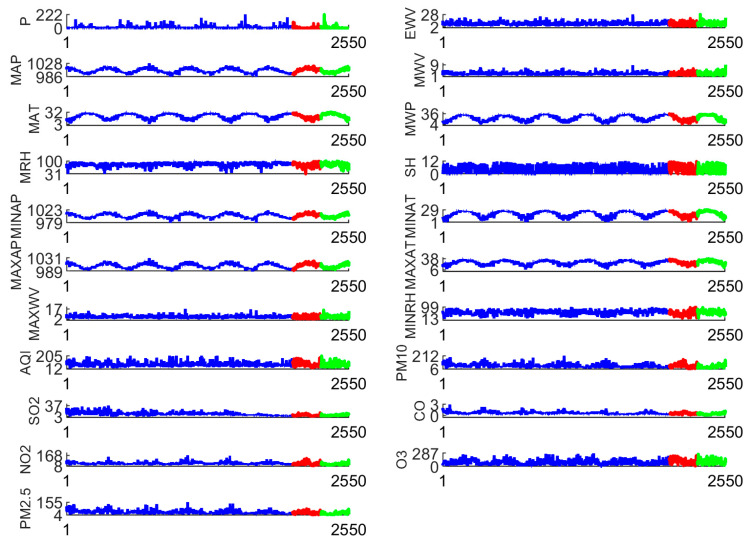
Meteorological and air pollution data of Guangzhou City. The blue line represents training data, the red line represents validation data, and the green line represents testing data.

Guangzhou City is the capital of Guangdong Province in China and a world-class city. The total area is about 7434.0 km^2^, with a population of 18.7341 million people and an urbanization rate of 86.48% in 2022. The regional GDP of Guangzhou is about 288,390 billion yuan. Air quality data and meteorological data are collected in Figure 1 and Table 1. The models need to be validated, so it is necessary to partition the raw data. 80% of the data were selected as the training set, 10% as the validation set, and the remaining 10% were used as the test set. In other words, these are divided into three groups: training set (from 1 January 2014 to 30 June 2019), validation set (from 1 July 2019 to 31 March 2020), and testing set (from 1 April 2020 to 31 December 2020).

### 2.2. Wavelet Transformation (WT)

WT is a multi-resolution data analysis method for original signal processing to extract useful frequency information. Compared to Fourier transformation (FT), wavelet transformation (WT) has better time-frequency analysis capability. WT can reveal hidden, detailed information and realize multi-scale decomposition of the original data through the translation of the wavelet function [63,64]. Owing to the simplicity of discrete WT (DWT), DWT is a more common technique in many studies than continuous WT (CWT). DWT can improve the forecast accuracy and reduce overfitting [65]. Therefore, the DWT is used to attain features of the original signals. Decomposition algorithms can reduce the volatility of the original signals and improve the recognition ability of deep learning models for the original signals.

The DWT is used to analyze meteorological factors and air pollutants in this research. The DWT continuously decomposes raw data through high-pass and low-pass filters to obtain high-frequency components (detailed coefficients, CD) and low-frequency components (approximation coefficients, CA) [66]. The DWT of the original data *f*(*t*) is calculated by

(1) $$f(t)=\sum_{i=1}^{J}CD_{i}(t)+CA_{J}(t),$$

where *J* is the number of decomposition levels. DWT represents *f*(*t*) in terms of the sum of subseries, consisting of high-frequency detail signals *CD*_1_, *CD*_2_, and *D_J_* and a low-frequency approximation signal *CA_J_*.

### 2.3. Artificial Neural Network (ANN)

**Figure 2:**
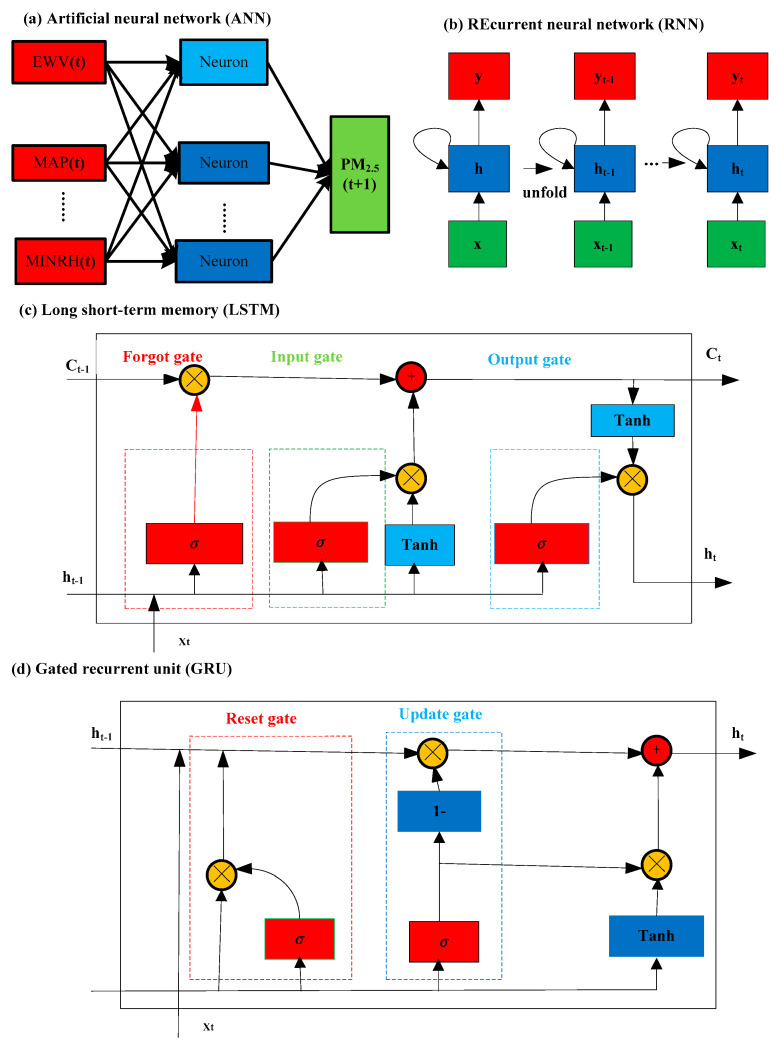
The architectures for predicting PM_2.5_ concentrations.

An ANN simulates the biological brain, and it is used to predict PM_2.5_ [15,67]. The ANN architecture consists of 3 layers, and each layer is composed of some artificial neurons (nodes) and an activation function (transfer function). Each node is contacted via weights and thresholds. The proposed ANN model for predicting PM_2.5_ concentrations is showcased in Figure 2a.

The activation functions (transfer functions) of the ANN and deep learning models are usually logarithmic sigmoid transfer function (*logsig*, or sigmoid), hyperbolic tangent activation function (*tansig*, or tanh), linear transfer function (*purelin*), and rectified linear units (*relu*, or poslin), respectively. The activation functions are calculated by

(2) $$logsig(b)=\frac{1}{1+e^{-b}},$$

(3) $$tansig(b)=\frac{e^{b}-e^{-b}}{e^{b}+e^{-b}},$$

(4) purelin(b)=b,

(5) relu(b)=max(0,b),

where *b* represents the corresponding input variable.

### 2.4. Recurrent Neural Network (RNN)

An RNN is a variant of ANN, and it can easily capture the temporal dynamic behavior of input (Figure 2b). Nodes (neurons) in an RNN have a “recursive” property [68]. An RNN has memory ability and can capture temporal dependencies. Therefore, it is suitable for PM_2.5_ time-series analysis.

### 2.5. Long Short-Term Memory (LSTM)

LSTM is an improved RNN, benefits from the advantages of the RNN technique, and utilizes the unique structure of gates to effectively solve the problem of gradient explosion and vanishing in the RNN. LSTM comprises several cyclic cells whose inputs include the input features of the current time, the state of network cells at the previous time, and the output of the hidden layer [69]. The LSTM structure is showcased in Figure 2c. *σ* represents a sigmoid function, Ct − 1 and *Ct* represent cell states, and ht − 1 and ht represent hidden states.

### 2.6. Gated Recurrent Unit (GRU)

The GRU is a variant of LSTM that maintains the predicted performance of LSTM and effectively solves the problems of gradient explosion and vanishing in RNN while simplifying the structure. The GRU has easier training and simpler construction than LSTM, and it can improve the efficiency of model training. The GRU only has two gates: the reset gate and the update gate. Compared to LSTM, it reduces one less and also reduces matrix multiplication operations. The GRU has fewer parameters and lower computational complexity. Therefore, the GRU takes less time while the magnitude of input data is large [69]. The structure of the GRU is showcased in Figure 2d.

### 2.7. Bidirectional Long Short-Term Memory (BiLSTM)

**Figure 3:**
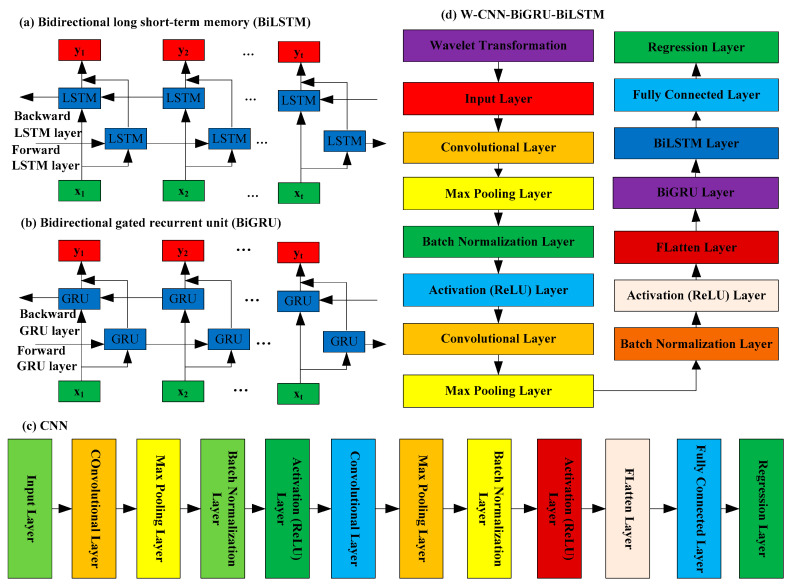
The network architectures of the proposed models.

BiLSTM is an evolution of LSTM based on Bidirectional RNN, an extension of RNN. BiLSTM consists of two LSTMs: forward and backward (Figure 3a). In the BiLSTM architecture, input information can be processed forward and backward. Information of t can use t − 1 and t + 1 information. Taken overall, BiLSTM is more effective and accurate than unidirectional LSTM [70].

### 2.8. Bidirectional Gated Recurrent Unit (BiGRU)

BiGRU consists of two unidirectional GRUs with opposite directions. BiGRU has an additional layer for hidden states [71]. The BiGRU architecture is shown in Figure 3b.

### 2.9. Convolutional Neural Network (CNN)

The CNN extracts map features from input data and reduces input data dimensionality, and the CNN is successfully applied in air pollution prediction (Figure 3c). The CNN includes convolution, pooling, fully connected, and regression layers [72]. The convolutional kernel, as the core component of a CNN, plays a crucial role in deep learning. The convolutional kernel performs convolution operations of input data through sliding windows to extract local features from the input data. The weights of the convolution kernel are shared. When using the same convolution kernel to perform convolution operations of different regions of the input data, the same weights are used. This parameter-sharing method greatly reduces the number of model parameters and improves the training efficiency of the CNN model. The pooling layer then extracts the most representative features from the obtained convolutional features, and it can reduce overfitting and dimensionality. The CNN is applied to PM_2.5_ time-series data prediction, and the convolution kernel moves only in the temporal direction, so it can extract correlations between local variables.

### 2.10. Hybrid Models

The complete integrated framework for better forecasting of daily PM_2.5_ concentrations is developed. Hybrid models include CNN-BiGRU, CNN-LSTM, CNN-GRU, CNN-BiLSTM, CNN-LSTM-GRU, CNN-GRU-LSTM, CNN-BiLSTM-BiGRU, CNN-BiGRU-BiLSTM, W-ANN, W-RNN, W-GRU, W-BiGRU, W-CNN, W-LSTM, W-BiLSTM, W-CNN-GRU, W-CNN-BiGRU, W-CNN-LSTM, W-CNN-BiLSTM, W-CNN-LSTM-GRU, W-CNN-GRU-LSTM, W-CNN-BiLSTM-BiGRU, and W-CNN-BiGRU-BiLSTM. By combining wavelet transform with ANN and deep learning, fifteen wavelet-based hybrid models are established. The W-CNN-BiGRU-BiLSTM model combines the advantages of the CNN model, the BiGRU model, the BiLSTM model, and the wavelet transformation technique. That is to say, it is a deep learning framework that combines the decomposition ability of wavelet transform, the powerful feature extraction ability of CNN, and the ability of time-series memory of BiGRU and BiLSTM for learning features related to PM_2.5_ concentrations from input variables. The model structure of W-CNN-BiGRU-BiLSTM is shown in Figure 3d. Firstly, wavelets decompose the raw data to obtain a series of sub-components; then, the CNN extracts features from these sub-components; finally, the BiGRU and BiLSTM utilize these features for PM_2.5_ time-series prediction.

The specific process is as follows:

(1) Feature selection: The correlation coefficient is used to discover the best input features that have the strongest relationship with PM_2.5_ concentration.
(2) Data decomposition: Wavelet functions are used to decompose input variables into high-frequency and low-frequency components.
(3) Combination prediction: Predict PM_2.5_ concentration using multiple deep learning models.
(4) Model evaluation: The prediction results of multiple models are evaluated using evaluation indices.

### 2.11. Normalization

The raw data were normalized between zero and one to acquire the minimal root mean square error (RMSE) values and the fast convergence of artificial intelligence models [48]. The formula is as follows:

(6) $$b_{j}^{\Delta }=\frac{b_{j}-b_{\min}}{b_{\max}-b_{\min}},$$

where $b_{j}^{\Delta }$ expresses the normalized data, $b_{\max}$ is the maximum value of the raw sequence, and $b_{\min}$ represents the minimum value of the raw sequence.

After model simulation, the forecasted values should be reversely normalized, and the formula is calculated by

(7) $$b_{j}=\left(b_{\max}-b_{\min}\right)b_{j}^{\Delta }+b_{\min}$$

### 2.12. Performance Criteria (Metrics)

In order to appraise the predicting performance of the artificial intelligence models, R, *RMSE*, MAPE, and MAE are defined using the following formulas [64]:

(8) $$R=\frac{\sum \left(G_{m}-\overset{-}{G})(K_{m}-\overset{-}{K}\right)}{\sqrt{\sum {\left(G_{m}-\overset{-}{G}\right)}^{2}{\left(K_{m}-\overset{-}{K}\right)}^{2}}},$$

(9) $$MAPE=\frac{1}{I}\sum \frac{\left|G_{m}-K_{m}\right|}{G_{m}}\times 100,$$

(10) $$RMSE=\sqrt{\frac{\sum {\left(G_{m}-K_{m}\right)}^{2}}{I}},$$

(11) $$MAE=\frac{1}{I}\sum \left|G_{m}-K_{m}\right|,$$

where G_m_ and K_m_ are, respectively, the values of observed and predicted PM_2.5_ data; and I is the length of original data. $\overset{-}{G}$ and $\overset{-}{K}$ are, respectively, the average of the observed and forecasted PM_2.5_ data.

MATLAB 2024 software is used to prepare the methods. Compared to methods that do not use wavelets, the computational burden of using wavelets increases threefold.

## 3. Results

### 3.1. Correlation Between Input Predictors and PM_2.5_

It is crucial to conduct an association study between input features and pollutants for a better prediction model. The concentrations of PM_2.5_ are influenced by various variables. Atmospheric temperature has a significant impact on the generation and diffusion of PM_2.5_. Precipitation can affect the wet deposition and chemical reactions of chemicals in the atmosphere. Atmospheric pressure affects atmospheric stability and vertical mixing. Wind speed and direction are crucial for the transmission and diffusion of PM_2.5_. Relative humidity affects the rate of chemical reactions and aerosol formation in the atmosphere. For instance, high wind speed helps to diffuse pollutant concentrations, high atmospheric pressure improves air quality, and high humidity deteriorates air quality [4,73,74]. Therefore, the characteristics of meteorological elements play an important role in air quality forecasting tasks [75]. AQI and other air pollutants also affect PM_2.5_ [76]. In order to select the suitable predictors, the R between the predictors and PM_2.5_ (t + 1) were calculated (Table 1). Here, t represents the current day, t − 1 represents the past day, and t + 1 represents the next day. The threshold of the absolute value of R is 0.15, and the significance level is 0.05. The order of the correlation coefficients of the elements in Table 1 is PM_2.5_ (t), PM_10_ (t), NO_2_ (t), SO_2_ (t), PM_2.5_ (t − 1), CO (t), AQI (t), PM_2.5_ (t − 2), MWP (t), MINAT (t), PM_2.5_ (t −3), MINRH (t), MAXAP (t), MAP (t), MINAP (t), PM_2.5_ (t − 4), MAT (t), PM_2.5_ (t − 5), EWV (t), MAXWV (t), MRH (t), PM_2.5_ (t − 6), MAXAT (t), P (t), O_3_ (t), MWV (t), and SH (t). PM_2.5_ (t + 1) had the highest correlation with PM_2.5_ (t), followed by PM_10_ (t), NO_2_ (t), and SO_2_ (t). Among meteorological elements, PM_2.5_ (t + 1) was most correlated with mean water pressure (t), followed by minimum atmospheric temperature (t) and minimum relative humidity (t). PM_2.5_ (t + 1) was positively correlated with mean atmospheric pressure (t), sunshine hours (t), minimum atmospheric pressure (t), and maximum atmospheric pressure (t), and negatively correlated with other meteorological factors. Overall, this analysis validated the substantial impact of meteorological factors on PM_2.5_ levels. Therefore, 27 predictors were selected as input features to the used models. PM_2.5_ (t + 1) was used as the output variable of the used models.

### 3.2. Selection of Mother Wavelets

**Figure 4:**
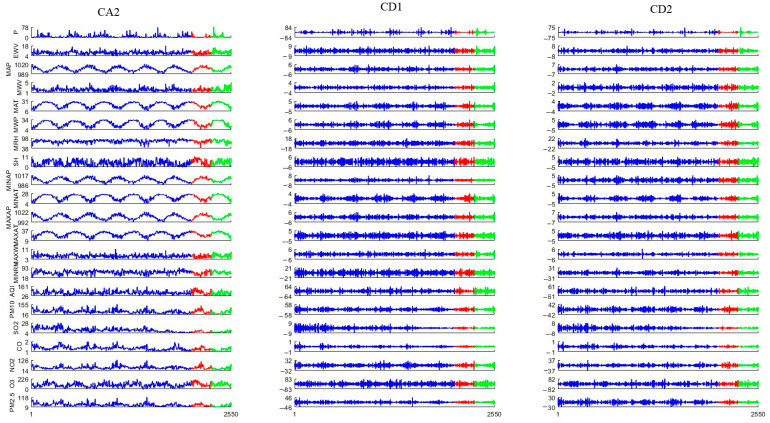
The result of wavelet decomposition. The blue line represents training data, the red line represents validation data, and the green line represents testing data.

The important information of input predictors is extracted by wavelet transformation. The selection of appropriate wavelet functions and decomposition scales affects the prediction results of PM_2.5_. Due to the decisive role of wavelet transformation in input signal feature extraction, it is necessary to select wavelet functions based on the required feature extraction. In general, input signals have different waveform characteristics at different scales. In addition, the spectra of input signals and noise are both nonlinear functions. Due to the unique characteristics of input signals and noise, they can be separated at different scales. Therefore, it is necessary to decompose input signals and noise separately at different scales. The various trends and details of input predictors are acquired by two-level wavelet decomposition. After two-level wavelet decomposition and reconstruction, the input predictors are decomposed into three sections. CA2 expresses the low-frequency information of the input predictors, and CD2 and CD1 express the high-frequency information of the input predictors. The variation characteristics of the input predictors are the key factors affecting mother wavelet selection [77]. The optimal wavelet function can be acquired through continuous iterative search and correlation coefficients [61]. The used mother wavelets are mainly symlets (sym), coiflets (coif), Daubechies (db), and biorthogonal wavelets (bior) [78]. Twenty-seven mother wavelets (wavelet functions) are used to decompose the raw data in order to select the appropriate wavelet function. The smaller the R between CA2, CD1, and CD2 of various wavelet functions, the better. From Table 2, it can be seen that the results of Bior1.1 were smallest, so Bior1.1 was chosen to decompose the original data. The wavelet function (Bior1.1) has good time-domain and frequency-domain characteristics, making it suitable for input signal feature extraction. The quantitative assessment revealed that the components of the input predictors were independent of one another. Figure 4 shows the decomposition results of the original signals using the wavelet function (Bior1.1). Each raw data point is decomposed into three components (approximative CA2, detailed CD1, and CD2). The decomposed components are more periodic and can better reflect the data characteristics of the original data. The raw data are decomposed to reduce their complexity. The fitting degree is significantly improved after wavelet decomposition.

### 3.3. Selection of the Hyperparameters in the Models

**Figure 5:**
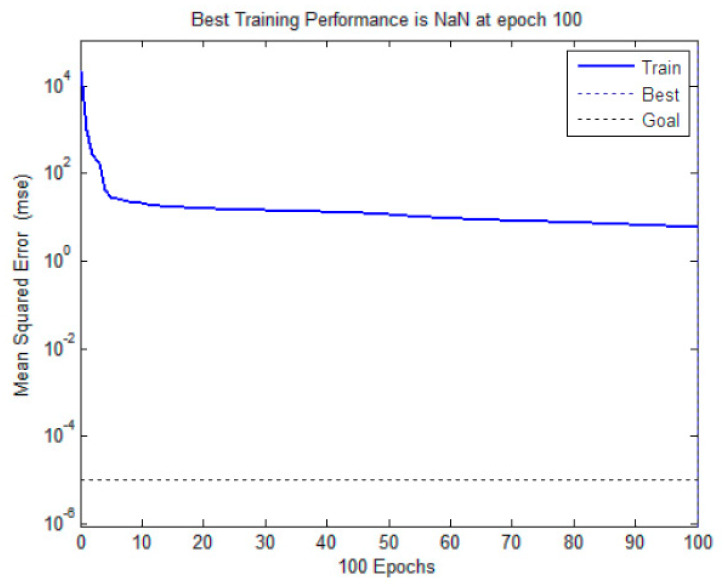
Learning curve convergence for the W-CNN-BiGRU-BiLSTM model.

A series of hyperparameters of the proposed ANN and deep learning models are ablated, including suitable learning rate, appropriate batch size, and good epochs. A grid search is used to optimize the parameters. Through model training and validation, the hyperparameters of the models are selected in Table 3. The hidden layer of the ANN has 21 neurons. The units of hidden layers in deep learning models are 100. The activation functions of the ANN are logsig and purelin. The activation functions of deep learning models are tanh and sigmoid, the number of epochs is 100, the learning rate is 0.001, the kernel size of the CNN is 3 × 1, the batch size is 15, the Convolution Filters are 16 and 32, Max-pooling is 2 × 1, and the Adam optimizer is utilized in deep learning models. The networks are trained and the parameters are updated using the adaptive moment estimation (Adam) optimizer. The Adam optimizer is used to improve the gradient descent method, which is conducive to the convergence of deep learning models [79]. The hyperparameters of the hybrid models are a combination of the parameters of the single models. The configuration of the W-CNN-BiGRU-BiLSTM model is as follows: the kernel size of the convolutional layer is 3 × 1, the Convolution Filters are 16 and 32, Max-pooling is 2 × 1, the units of hidden layers in BiGRU and BiLSTM are 100, the number of epochs is 100, the learning rate is 0.001, the batch size is 15, and the optimizer is Adam. Furthermore, the learning curve of the W-CNN-BiGRU-BiLSTM model is presented in Figure 5, where the MSE enables a comprehensive conclusion that the best model effectively learns.

### 3.4. Performance Comparison of the Various Models

The model performance indicators (R, RMSE, MAE, and MAPE) of ANN and deep learning-based forecasting techniques are given in Table 4. The performance of deep learning models was much better than that of the ANN. The hybrid models were significantly superior to the single deep learning predicting techniques for all periods. The hybrid models greatly improved the performance of the single models. This means that hybrid models were better able to capture the complex relationship between the predictors and PM_2.5_. For example, the performance of the CNN-LSTM model was much better than that of single LSTM and CNN. Compared to other models, The CNN-BiGRU-BiLSTM model had the highest R value and the lowest MAE value. The CNN-BiGRU-BiLSTM outperformed other models by comparing all levels (R, MAPE, RMSE, and MAE) during the training, validation, and predicting periods. The RMSE values of CNN-BiGRU-BiLSTM are, respectively, 9.0623, 8.8398, and 7.6418 μg/m^3^ during the training, validation, and predicting stages. Nevertheless, the corresponding RMSE values are, respectively, 10.7041, 11.6762, and 10.3658 μg/m^3^ for CNN; 11.4148, 12.1985, and 11.3294 μg/m^3^ for BiGRU; and 9.9673, 10.6220, and 9.5961 μg/m^3^ for BiLSTM. Similarly, the MAPE values of CNN-BiGRU-BiLSTM are, respectively, 25.7337%, 27.4619%, and 26.2684% during the training, validation, and predicting periods. However, the corresponding MAPE values are, respectively, 29.8351%, 45.0852%, and 48.2678% for CNN; 30.3307%, 45.3682%, and 52.7421% for BiGRU; and 28.5275%, 37.6773%, and 39.7882% for BiLSTM. It is indicated that the employed CNN-BiGRU-BiLSTM could achieve the best simulation performance in all evaluation criteria (R, RMSE, MAE, and MAPE) when compared with other deep learning models.

Similarly, the model performance criteria of W-ANN and wavelet-based deep learning models are given in Table 5. The performance of wavelet-based deep learning models was much better than that of the W-ANN. The proposed W-CNN-BiGRU-BiLSTM model obtained the best simulation performance in all evaluation criteria (R, RMSE, MAE, and MAPE) during the training, validation, and predicting phases. The W-CNN-BiGRU-BiLSTM model had the highest R value and the lowest MAE, RMSE, and MAPE values. For example, the RMSE values of W-CNN-BiGRU-BiLSTM are, respectively, 1.9301, 1.4933, and 1.4935 μg/m^3^ during the training, validation, and predicting stages. Nevertheless, the corresponding RMSE values are, respectively, 8.8393, 7.4215, and 7.5615 μg/m^3^ for W-CNN; 8.9401, 8.4798, and 8.6062 μg/m^3^ for W-BiGRU; and 7.3921, 7.0324, and 6.7973 μg/m^3^ for W-BiLSTM. As for the MAPE, the MAPE values of W-CNN-BiGRU-BiLSTM are, respectively, 4.8861%, 5.2563%, and 7.3782% during the training, validation, and predicting stages. Nevertheless, the corresponding MAPE values are, respectively, 19.3726%, 19.2812%, and 28.6538% for W-CNN; 19.6657%, 22.5931%, and 31.4817% for W-BiGRU; and 18.3820%, 18.1092%, and 18.4883% for W-BiLSTM. More importantly, the W-CNN-BiGRU-BiLSTM achieved better prediction performance than W-CNN-LSTM-GRU, W-CNN-GRU-LSTM, and W-CNN-BiLSTM-BiGRU. In particular, the MAPE of the W-CNN-BiGRU-BiLSTM acquired a more accurate forecast value than the corresponding MAPE values among all the other fourteen wavelet-based deep learning forecasting models. Therefore, it has been proven that the proposed W-CNN-BiGRU-BiLSTM model can accomplish relatively stable and more accurate PM_2.5_ prediction.

As shown in Table 4 and Table 5, the prediction errors of wavelet-based deep learning models were lower than other deep learning models. By using secondary DWT, the sequence of input variables is decomposed into three subsequences with good stationarity for model training. This facilitates the extraction of mainstream components, thereby significantly improving prediction performance. Through a comprehensive comparison of thirty models, wavelet preprocessing can help improve the prediction of daily PM_2.5_ concentrations. The W-LSTM method outperformed the existing LSTM method in predicting PM_2.5_ concentrations. The W-CNN-BiGRU-BiLSTM model had the best performance by using various evaluation criteria. In addition, the W-ANN was superior to the ANN. Compared to the ANN, the RMSE, MAE, and MAPE of the hybrid W-CNN-BiGRU-BiLSTM prediction model decreased by 87.52%, 87.80%, and 88.24%, respectively. Compared with the LSTM prediction model, the RMSE of the hybrid W-CNN-BiGRU-BiLSTM model decreased by 71.39%, the MAE decreased by 70.27%, and the MAPE decreased by 61.01%. The performance of the W-CNN-BiGRU-BiLSTM model was much better than CNN-BiGRU-BiLSTM. Similarly, the prediction accuracy of the W-CNN-BiLSTM model was higher than CNN-BiLSTM. The prediction error of the W-CNN-GRU-LSTM model was much smaller than CNN-GRU-LSTM. The R values of the W-BiLSTM model were much higher than BiLSTM. Compared to the deep learning prediction models, the wavelet-based deep learning models could better predict nonlinear and non-stationary PM_2.5_ concentrations. The consistency of these results demonstrated the effectiveness of the proposed wavelet-based hybrid prediction models in improving the prediction accuracy of PM_2.5_ concentrations in Guangzhou City. The prediction results of all wavelet-based deep learning models are within the acceptable range. The experimental results show that wavelet transform can effectively improve the prediction accuracy of deep learning, further demonstrating the effectiveness of the combined W-CNN- BiGRU-BiLSTM model proposed in this paper.

**Figure 6:**
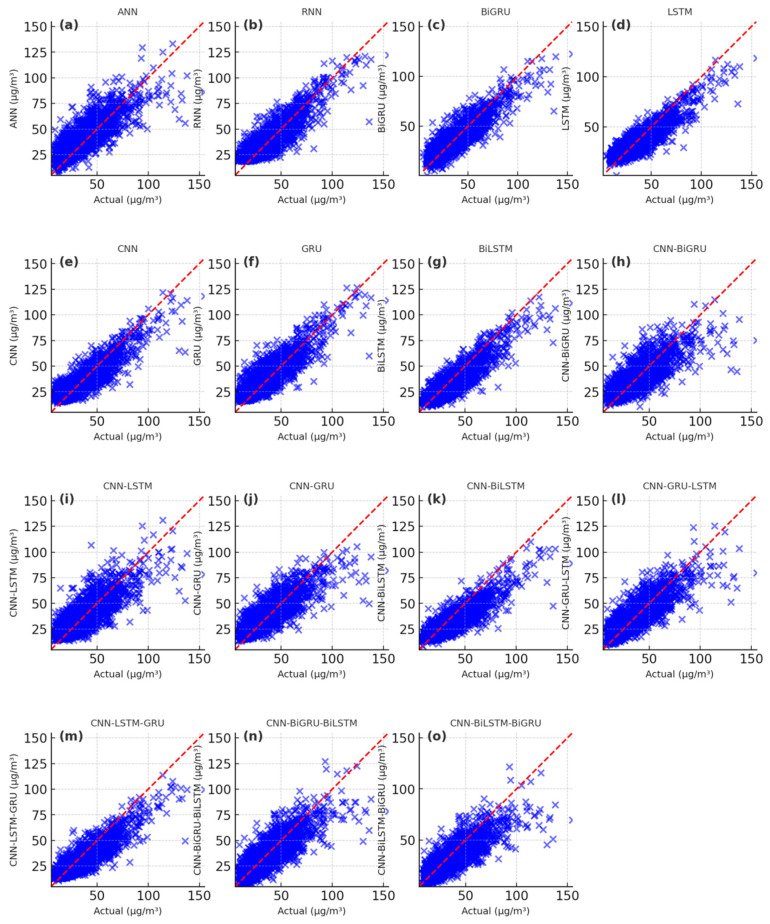
Scatter plot of the simulated PM_2.5_ results during the training period with the deep learning models. (**a**) the simulated results of ANN, (**b**) the simulated results of RNN, (**c**) the simulated results of BiGRU, (**d**) the simulated results of LSTM, (**e**) the simulated results of CNN, (**f**) the simulated results of GRU, (**g**) the simulated results of BiLSTM, (**h**) the simulated results of CNN-BiGRU, (**i**) the simulated results of CNN-LSTM, (**j**) the simulated results of CNN-GRU, (**k**) the simulated results of CNN-BiLSTM, (**l**) the simulated results of CNN-GRU-LSTM, (**m**) the simulated results of CNN-LSTM-GRU, (**n**) the simulated results of CNN-BiGRU-BiLSTM, (**o**) the simulated results of CNN-BiLSTM-BiGRU.

**Figure 7:**
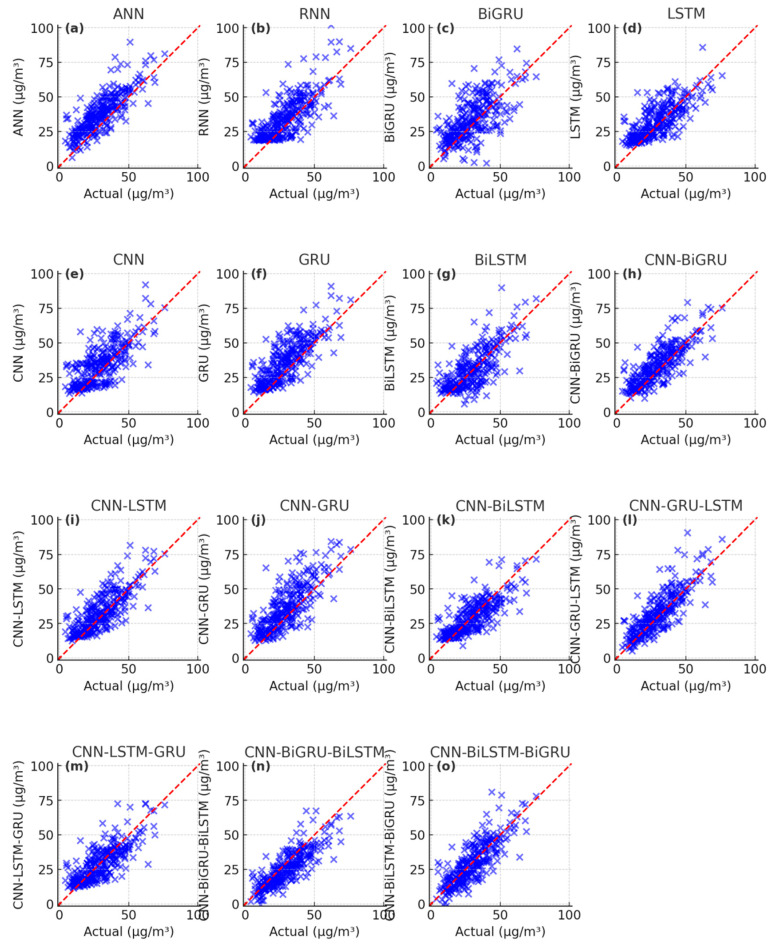
Scatter plot of the simulated PM_2.5_ results during the validation period with the deep learning models. (**a**) the simulated results of ANN, (**b**) the simulated results of RNN, (**c**) the simulated results of BiGRU, (**d**) the simulated results of LSTM, (**e**) the simulated results of CNN, (**f**) the simulated results of GRU, (**g**) the simulated results of BiLSTM, (**h**) the simulated results of CNN-BiGRU, (**i**) the simulated results of CNN-LSTM, (**j**) the simulated results of CNN-GRU, (**k**) the simulated results of CNN-BiLSTM, (**l**) the simulated results of CNN-GRU-LSTM, (**m**) the simulated results of CNN-LSTM-GRU, (**n**) the simulated results of CNN-BiGRU-BiLSTM, (**o**) the simulated results of CNN-BiLSTM-BiGRU.

**Figure 8:**
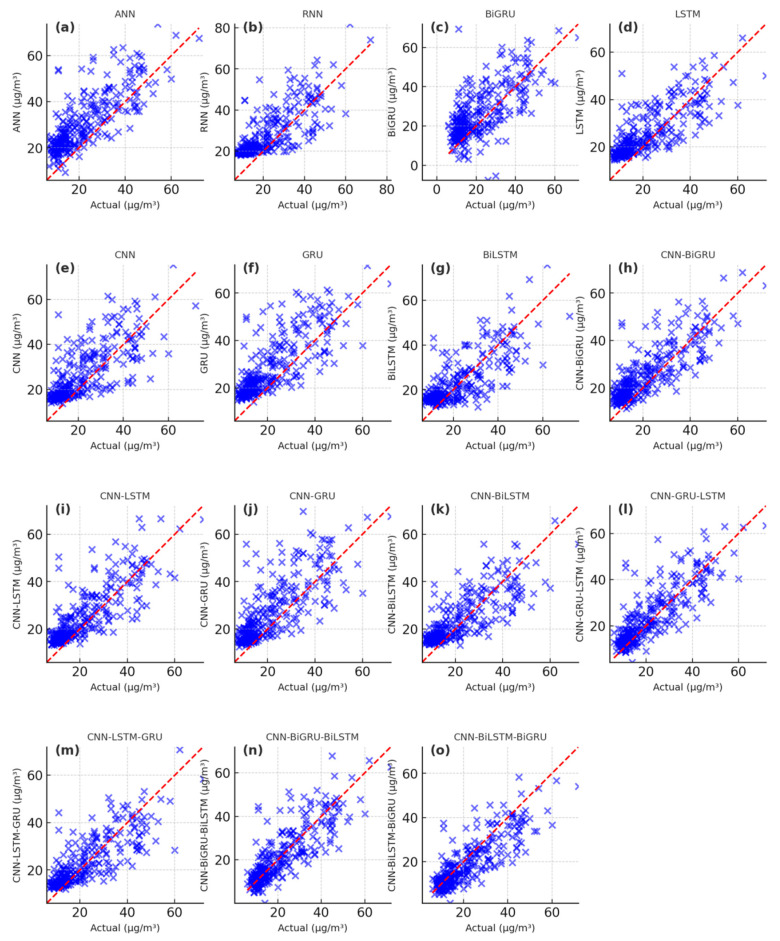
Scatter plot of the predicting PM_2.5_ results during the predicting period with the deep learning models. (**a**) the predicting results of ANN, (**b**) the predicting results of RNN, (**c**) the predicting results of BiGRU, (**d**) the predicting results of LSTM, (**e**) the predicting results of CNN, (**f**) the predicting results of GRU, (**g**) the predicting results of BiLSTM, (**h**) the predicting results of CNN-BiGRU, (**i**) the predicting results of CNN-LSTM, (**j**) the predicting results of CNN-GRU, (**k**) the predicting results of CNN- BiLSTM, (**l**) the predicting results of CNN-GRU-LSTM, (**m**) the predicting results of CNN-LSTM-GRU, (**n**) the predicting results of CNN-BiGRU-BiLSTM, (**o**) the predicting results of CNN-BiLSTM-BiGRU.

The simulating results of PM_2.5_ concentrations using the deep learning models are presented in Figure 6, Figure 7 and Figure 8. The scatter plots of the ANN model exhibit high bias, with loosely distributed points that overestimate PM_2.5_ values, demonstrating weak capability in capturing nonlinear temporal relationships (Figure 6a, Figure 7a, Figure 8a). Similarly, the RNN, BiGRU, LSTM, CNN, GRU, CNN-GRU, CNN-BiGRU, CNN-LSTM, and CNN-BiLSTM models also overestimate PM_2.5_ values (Figure 6b–k and Figure 8b–k). In contrast, the CNN-LSTM-GRU, CNN-GRU-LSTM, CNN-BiLSTM-BiGRU, and CNN-BiGRU-BiLSTM models show scatter points closely aligned along the diagonal (Figure 6i–o, Figure 7i–o, Figure 8i–o). Among these, the CNN-BiGRU-BiLSTM model’s scatter points are the closest to the diagonal. The simulating performance of the CNN-BiGRU-BiLSTM method was superior to other deep learning models during the training, validation, and prediction stages. Although all deep learning models could predict the long-term future trend of PM_2.5_ concentrations, the proposed CNN-BiGRU-BiLSTM method could better fit the raw PM_2.5_ concentration curve.

**Figure 9:**
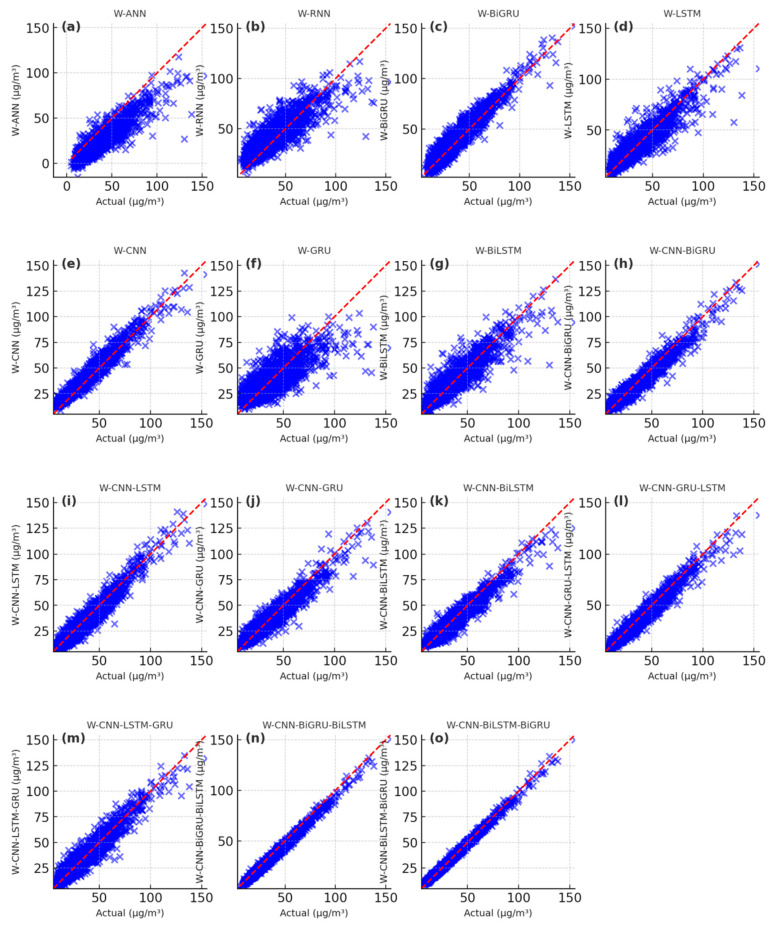
Scatter plot of the simulated PM_2.5_ results during the training period with the wavelet-based deep learning models. (**a**) the simulated results of W-ANN, (**b**) the simulated results of W-RNN, (**c**) the simulated results of W-BiGRU, (**d**) the simulated results of W-LSTM, (**e**) the simulated results of W-CNN, (**f**) the simulated results of W-GRU, (**g**) the simulated results of W-BiLSTM, (**h**) the simulated results of W-CNN-BiGRU, (**i**) the simulated results of W-CNN-LSTM, (**j**) the simulated results of W-CNN-GRU, (**k**) the simulated results of W-CNN- BiLSTM, (**l**) the simulated results of W-CNN-GRU-LSTM, (**m**) the simulated results of W-CNN-LSTM-GRU, (**n**) the simulated results of W-CNN-BiGRU-BiLSTM, (**o**) the simulated results of W-CNN-BiLSTM-BiGRU.

**Figure 10:**
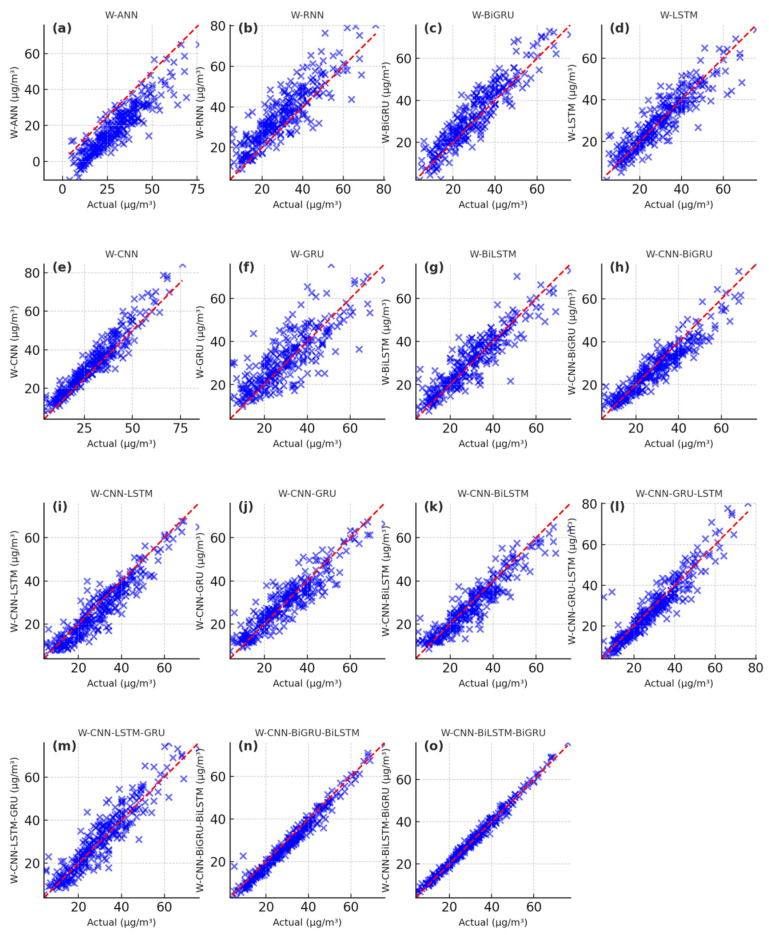
Scatter plot of the simulated PM_2.5_ results during the validation period with the wavelet-based deep learning models. (**a**) the simulated results of W-ANN, (**b**) the simulated results of W-RNN, (**c**) the simulated results of W-BiGRU, (**d**) the simulated results of W-LSTM, (**e**) the simulated results of W-CNN, (**f**) the simulated results of W-GRU, (**g**) the simulated results of W-BiLSTM, (**h**) the simulated results of W-CNN-BiGRU, (**i**) the simulated results of W-CNN-LSTM, (**j**) the simulated results of W-CNN-GRU, (**k**) the simulated results of W-CNN-BiLSTM, (**l**) the simulated results of W-CNN-GRU-LSTM, (**m**) the simulated results of W-CNN-LSTM-GRU, (**n**) the simulated results of W-CNN-BiGRU-BiLSTM, (**o**) the simulated results of W-CNN-BiLSTM-BiGRU.

**Figure 11:**
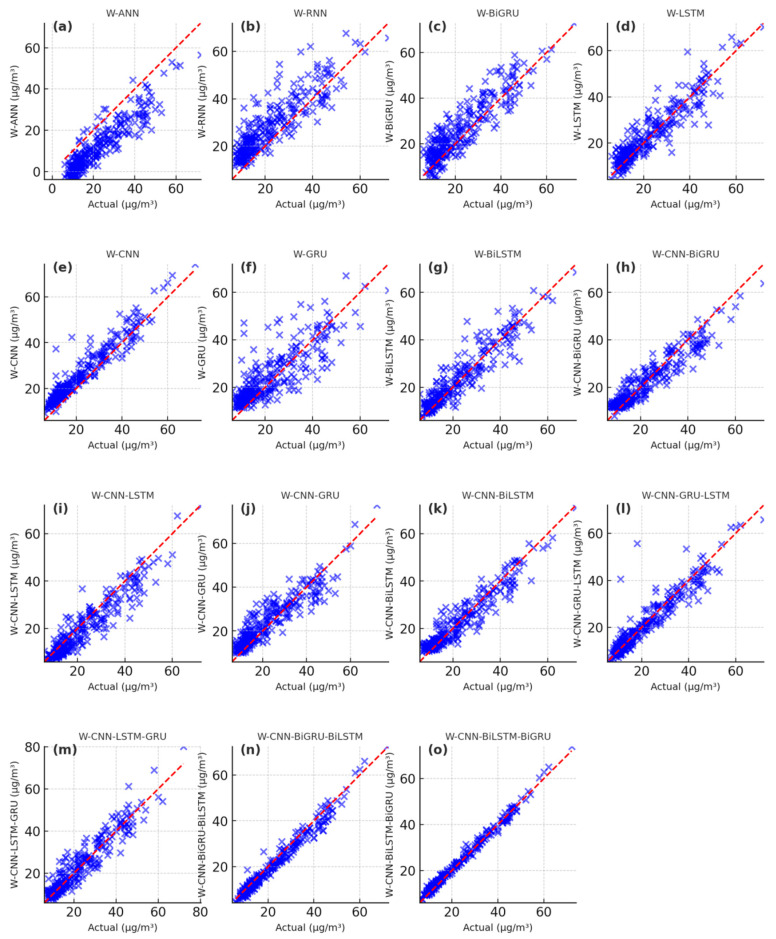
Scatter plot of the predicting PM_2.5_ results during the predicting period with the wavelet-based deep learning models. (**a**) the predicting results of W-ANN, (**b**) the predicting results of W-RNN, (**c**) the predicting results of W-BiGRU, (**d**) the predicting results of W-LSTM, (**e**) the predicting results of W-CNN, (**f**) the predicting results of W-GRU, (**g**) the predicting results of W-BiLSTM, (**h**) the predicting results of W-CNN-BiGRU, (**i**) the predicting results of W-CNN-LSTM, (**j**) the predicting results of W-CNN-GRU, (**k**) the predicting results of W-CNN-BiLSTM, (**l**) the predicting results of W-CNN-GRU-LSTM, (**m**) the predicting results of W-CNN-LSTM-GRU, (**n**) the predicting results of W-CNN-BiGRU-BiLSTM, (**o**) the predicting results of W-CNN-BiLSTM-BiGRU.

The PM_2.5_ fitting and testing results of wavelet-based deep learning models are showcased in Figure 9, Figure 10 and Figure 11. The scatter plots of the W-ANN, W-RNN, and W-BiGRU models display bias and loosely distributed points (Figure 9a–c, Figure 10a–c, Figure 11a–c), while the W-CNN, W-LSTM, and W-GRU models also exhibit underestimation of peak values, particularly in long-term predictions (Figure 9d–f, Figure 10d–f, Figure 11d–f). The W-BiLSTM model’s scatter plots fit the true values better than W-GRU but still show some lag (Figure 9g, Figure 10g, Figure 11g). The W-CNN-GRU, W-CNN-BiGRU, W-CNN-LSTM, and W-CNN-BiLSTM models demonstrate smoothed local fluctuations but suffer from underfitting in long-term trends (Figure 9h–k, Figure 10h–k, Figure 11h–k). On the other hand, the W-CNN-LSTM-GRU, W-CNN-GRU-LSTM, W-CNN-BiLSTM-BiGRU, and W-CNN-BiGRU-BiLSTM models exhibit scatter points tightly clustered around the diagonal, with smaller errors in high-value regions (Figure 9i–o, Figure 10i–o, Figure 11i–o). The W-CNN-BiLSTM-BiGRU model is more stable than W-CNN-LSTM-GRU and more sensitive to sudden rises/drops in PM_2.5_, especially in long-term predictions (Figure 9n, Figure 10n, Figure 11n). The W-CNN-BiGRU-BiLSTM model’s scatter points are more symmetric and closer to the diagonal compared to W-CNN-BiLSTM-BiGRU, demonstrating the best overall performance, the most concentrated error distribution, and higher accuracy in predicting abrupt changes and sustained high PM_2.5_ intervals (Figure 9o, Figure 10o, Figure 11o). In summary, by analyzing the distribution patterns, error concentration, and the ability to capture extreme PM_2.5_ values in these scatter plots, we can intuitively assess the simulation and predictive capabilities of different models.

To our surprise, the hybrid models performed very well in reproducing dynamic features. The simulating performance of the W-CNN-BiGRU-BiLSTM method was superior to other deep learning models during the three stages. Moreover, the figures showcase clearly that the overall performance of W-CNN-BiLSTM-BiGRU and W-CNN-BiGRU-BiLSTM were superior to the W-CNN and W-BiGRU models during the training, validation, and prediction stages. The trends of the W-CNN-BiLSTM-BiGRU and W-CNN-BiGRU-BiLSTM models had better agreement with observations than the W-CNN and W-BiGRU models. It is indicated that the W-CNN-BiGRU-BiLSTM model performed satisfyingly in the three stages. The trend of the W-CNN-BiGRU-BiLSTM prediction result was closely accordant with the observed PM_2.5_ concentration data.

## 4. Discussion

For accurately predicting PM_2.5_ concentrations in Guangzhou City, different machine learning and wavelet-based deep learning models were proposed to forecast PM_2.5_ in Guangzhou City. The performance of the methods was compared by RMSE, MAE, MAPE, and R. The W-CNN-BiGRU-BiLSTM model outperforms the other models. The W-CNN-BiGRU-BiLSTM exhibited significant advantages over other models in processing input factors. The W-CNN-BiGRU-BiLSTM method combines wavelet (W), CNN, BiLSTM, and BiGRU. Wavelet transformation is applied to decompose the meteorological and air pollutant data into sub-level sequences. The CNN algorithm is good at extracting local and spatial features. The BiLSTM and the BiGRU can better handle the bidirectional dependencies of sequence data while considering forward and backward contextual information, thereby improving the accuracy of PM_2.5_ prediction. Correspondingly, simulation outcomes also indicated that the W-CNN-BiGRU-BiLSTM can significantly improve the predictive performance compared to other deep learning models. The wavelet transform technique plays an important role in enhancing the PM_2.5_ prediction ability and accuracy of the deep learning (DL) prediction model. From the above results, the wavelet-based deep learning models outperform the deep learning models in predicting PM_2.5_ concentrations.

Wavelet transforms are powerful tools for signal processing, allowing for multi-resolution analysis. In the context of neural networks, wavelets can be used for preprocessing or feature extraction, enabling the capture of both frequency and time-domain characteristics in input signals. This preprocessing step can significantly enhance the performance of deep learning models when applied to PM_2.5_ time-series data or sequences. LSTM, GRU, and their bidirectional counterparts excel in PM_2.5_ sequence-based tasks due to their ability to capture long-term dependencies.

Hybrid models combine CNNs with other neural networks to leverage the strengths of both architectures. For instance, CNN-LSTM combines CNN’s feature extraction capabilities with LSTM’s ability to capture temporal dependencies, providing a powerful tool for PM_2.5_ time-series forecasting. Other hybrid models, such as CNN-BiLSTM or CNN-BiGRU, enhance the capability to process sequential data by considering both past and future contexts while also extracting rich features through convolutional layers. These more complex hybrid architectures further combine multiple models to capture various levels of features and temporal dependencies. For example, CNN-LSTM-GRU could be used in scenarios requiring both short-term and long-term memory with feature extraction. Hybrid models improve performance further by combining the feature extraction capabilities of CNN with the sequential learning power of GRU and LSTM. CNN-LSTM-GRU provides flexibility in handling various types of input data, making it suitable for PM_2.5_ time-series forecasting. The intricate architectures like CNN-BiLSTM-BiGRU offer multi-layered mechanisms to process data from both directions (BiLSTM, BiGRU) while extracting features through CNN layers, optimizing performance on PM_2.5_ prediction tasks with complex patterns and dependencies.

Accurate PM_2.5_ prediction is crucial for providing critical information for policy decisions and public health. By providing reliable PM_2.5_ predictions, wavelet-based deep learning models can help stakeholders develop targeted strategies to improve air quality and safeguard public health. In summary, this study demonstrates the effectiveness of wavelet deep learning models in PM_2.5_ prediction and provides valuable insights into optimization strategies for improving model performance. By addressing key hyperparameters, we can improve our understanding of PM_2.5_ prediction. Based on the predicted results, reference data can be provided for urban planning, land uses, and spatial structures to minimize PM_2.5_ emissions.

Although our models have achieved good predictive performance, there are several noteworthy limitations of the wavelet-based deep learning models for PM_2.5_ prediction in Guangzhou City. Firstly, the prediction accuracy of the prediction models is closely related to input parameters, such as meteorological factors and other air pollutants. Due to the dependence of model development on the specific meteorological conditions of Guangzhou City, the model may not be able to capture the complexity of all environmental factors that affect PM_2.5_ concentrations. In addition, model development is based on daily average data. The lack of real-time hourly meteorological data and pollution emission data in Guangzhou City may affect the accuracy of the proposed model [80]. Thirdly, the lack of external pollution inputs from other regions may lead to the neglect of potential sources of pollution [81]. Additionally, we do not consider the impact of different seasons of El Niño and El Niño on PM_2.5_ concentrations [82]. Fourthly, optimizing all the hyperparameters of each model can be a time-consuming process, so we only considered the common hyperparameters. However, the wavelet-based deep learning model will be widely applied. By incorporating supplementary input variables, such as real-time meteorological data and hourly data from pollution sources and other regions, the accuracy and reliability of the wavelet-based deep learning model can be significantly improved. These data can be obtained through meteorological stations and air quality monitoring stations, which will greatly improve the accuracy and comprehensiveness of the hybrid model. Finally, integrating wavelet-based deep learning models with chemical transport models could improve the accuracy of PM_2.5_ prediction.

## 5. Conclusions

Wavelet-based deep learning methods for predicting PM_2.5_ concentrations in Guangzhou from 2014 to 2020 were developed and compared. The input variables were derived from the correlation coefficients between PM_2.5_ and predictors, including meteorological factors and air pollutants. Second-level discrete wavelet transformation (DWT) was used to preprocess the raw data and decompose it into three different frequency subsequences. Fifteen wavelet-based hybrid models were compared with fifteen machine learning models using the same historical data and a validation mechanism. When multiple frequency subsequences were utilized as inputs, the prediction accuracy of PM_2.5_ concentrations was significantly improved. Further, the findings showed the potential of the wavelet-based hybrid models in forecasting PM_2.5_ concentrations. The W-CNN-BiGRU-BiLSTM model demonstrated the highest predictive accuracy for PM_2.5_ levels. This proposed W-CNN-BiGRU-BiLSTM method successfully outperformed the benchmark deep learning methods (CNN, BiGRU, and BiLSTM). For the 1-day-ahead forecasting, the proposed W-CNN-BiGRU-BiLSTM model showed an RMSE advantage of 85.59%, 86.82%, and 84.44%, respectively, over the CNN, BiGRU, and BiLSTM models in Guangzhou. The W-CNN-BiGRU-BiLSTM model provided more accurate results. The W-CNN-BiGRU-BiLSTM model approached the W-CNN-BiLSTM-BiGRU model in some individual coefficients, but the relative error distribution indicated that the W-CNN-BiGRU-BiLSTM model had more stable performance. Also, the performance of deep learning models is much better than that of the ANN. Additionally, the prediction accuracy of hybrid models is higher than that of the single models. Moreover, the prediction errors of wavelet-based hybrid models are lower than for other hybrid models. The proposed hybrid W-CNN-BiGRU-BiLSTM model is a promising and practical method for PM_2.5_ prediction. It should be emphasized that the detailed information about the deep learning models and parameter setting rules for the wavelet transformation technique in this research can offer a useful reference for future study. Overall, air pollution prediction models can contribute to sustainable development and to improving people’s health, and they can provide information for government agencies to formulate air pollution prevention and control strategies.

In the future, we will apply this method to other cities to verify its effectiveness. Meanwhile, a multi-lead time predicting model of daily PM_2.5_ concentrations will be studied and developed. PM_2.5_ has certain differences in space and time, but it is feasible to use meteorological elements and pollutants as the input factors of the model in prediction and analysis. The addition of input parameters as predictors can improve the predicting model performance. Other factors need to be considered, including building height, terrain, traffic data, pollutant emissions, vegetation, transportation, land use, population, GDP, etc. New hybrid models will be utilized, such as wavelet transform and transformer, graph convolutional neural (GCN), transfer learning, generative adversarial network (GAN), etc.

## Figures and Tables

**Table 1:** The Pearson correlation coefficients (R) between PM_2.5_ (t + 1) and input predictors used for the model training.

Influence Factor	Abbreviation	R
Precipitation (t)	P (t)	−0.2021
Extreme wind velocity (t)	EWV (t)	−0.3554
Mean atmospheric pressure (t)	MAP (t)	0.4027
Mean wind velocity (t)	MWV (t)	−0.1720
Mean atmospheric temperature (t)	MAT (t)	−0.3696
Mean water pressure (t)	MWP (t)	−0.4440
Mean relative humidity (t)	MRH (t)	−0.3034
Sunshine hours (t)	SH (t)	0.1549
Minimum atmospheric pressure (t)	MINAP (t)	0.4024
Minimum atmospheric temperature (t)	MINAT (t)	−0.4373
Maximum atmospheric pressure (t)	MAXAP (t)	0.4084
Maximum atmospheric temperature (t)	MAXAT (t)	−0.2348
Maximum wind velocity (t)	MAXWV (t)	−0.3388
Minimum relative humidity (t)	MINRH (t)	−0.4134
AQI (t)	AQI (t)	0.4978
PM_10_ (t)	PM_10_ (t)	0.7203
SO_2_ (t)	SO_2_ (t)	0.5717
CO (t)	CO (t)	0.5119
NO_2_ (t)	NO_2_ (t)	0.6166
O_3_ (t)	O_3_ (t)	0.1748
PM_2.5_ (t)	PM_2.5_ (t)	0.7507
PM_2.5_ (t − 1)	PM_2.5_ (t − 1)	0.5577
PM_2.5_ (t − 2)	PM_2.5_ (t − 2)	0.4705
PM_2.5_ (t − 3)	PM_2.5_ (t − 3)	0.4306
PM_2.5_ (t − 4)	PM_2.5_ (t − 4)	0.3871
PM_2.5_ (t − 5)	PM_2.5_ (t − 5)	0.3511
PM_2.5_ (t − 6)	PM_2.5_ (t − 6)	0.3028

**Table 2:** R between CD1, CA2, and CD2 for the wavelet bases.

Mother Wavelets	CA2 and CD1	CA2 and CD2	CD1 and CD2
db2	−0.0009	0.9643	0.0006
db3	0.0015	−0.0012	0.0017
db4	0.0005	−0.0005	0.0006
db5	−0.0012	0.0026	−0.001
db6	−0.0019	0.0022	−0.0014
db7	−0.0015	0.0015	0.0001
db8	0.0003	0.0008	0.0003
db9	0.0015	−0.0005	0.0017
db10	0.002	−0.0014	0.0022
sym2	0.0015	−0.0009	0.0006
sym3	0.0015	−0.0012	0.0017
sym4	−0.0014	0.0018	−0.0012
sym5	0.0001	0.0001	0.0006
sym6	−0.0014	0.0017	−0.0016
sym7	0.0015	−0.0006	0.0027
sym8	−0.0018	0.0019	−0.0009
coif1	−0.0016	0.002	−0.0012
coif2	−0.002	0.0021	0.9153
coif3	−0.0022	0.0019	−0.0009
coif4	−0.0021	0.0022	−0.0014
coif5	−0.0021	0.002	−0.0016
bior1.1	0	0	0
bior2.2	0.0029	0.0143	0.0169
bior3.3	0.0005	−0.061	0.0006
bior4.4	−0.0011	−0.0002	0.0098
bior5.5	0.0033	−0.0122	0.0002
bior6.8	−0.0016	0.0013	0.0024

**Table 3:** The hyperparameters of the single models.

Hyperparameters	ANN	RNN	GRU	BiGRU	LSTM	BiLSTM	CNN
Units in hidden layer	21	100	100	100	100	100	
Activation function	logsig-purelin	tanh-sigmoid					Relu
Learning rate	0.001	0.001	0.001	0.001	0.001	0.001	0.001
Batch size	15	15	15	15	15	15	15
Epochs	100	100	100	100	100	100	100
Optimizer	Trainbr	Adam	Adam	Adam	Adam	Adam	Adam
Kernel size							3 × 1
Max-pooling							2 × 1
Convolution Filters							16–32

**Table 4:** Performance comparison of various deep learning models for simulating PM_2.5_ concentrations during the predicting phase.

Models	R	RMSE (μg/m^3^)	MAE (μg/m^3^)	MAPE (%)
ANN	0.6630	11.9688	9.9072	62.7491
RNN	0.6779	11.3927	9.7002	54.7036
GRU	0.6793	11.3511	8.9722	54.2364
BiGRU	0.6802	11.3294	8.8436	52.7421
CNN	0.7330	10.3658	8.2465	48.2678
LSTM	0.7458	10.0380	8.1713	45.6636
BiLSTM	0.7609	9.5961	7.6862	39.7882
CNN-GRU	0.7426	10.1655	8.1557	47.2628
CNN-BiGRU	0.7580	9.6270	7.7992	41.7416
CNN-LSTM	0.7810	8.5610	7.5092	38.9337
CNN-BiLSTM	0.7856	8.2600	6.6141	37.7028
CNN-LSTM-GRU	0.8005	7.8311	5.8929	31.9232
CNN-GRU-LSTM	0.8123	7.8235	5.7128	31.1673
CNN-BiLSTM-BiGRU	0.8183	7.8196	5.6382	29.0080
CNN-BiGRU-BiLSTM	0.8323	7.6418	5.5519	26.2684

**Table 5:** Performance comparison of various wavelet-based deep learning models for simulating PM_2.5_ concentrations during the predicting phase.

Models	R	RMSE (μg/m^3^)	MAE (μg/m^3^)	MAPE (%)
W-ANN	0.8188	11.5113	9.5490	58.4057
W-RNN	0.8718	8.8379	7.2191	44.6752
W-GRU	0.8890	8.7971	6.8600	34.1093
W-BiGRU	0.9029	8.6062	6.2471	31.4817
W-CNN	0.9161	7.5615	5.5796	28.6538
W-LSTM	0.9133	7.2930	5.0708	20.6160
W-BiLSTM	0.9223	6.7973	4.4111	18.4883
W-CNN-GRU	0.9122	7.3323	5.0871	20.6178
W-CNN-BiGRU	0.9212	7.2920	4.4431	18.7684
W-CNN-LSTM	0.9344	6.6869	3.6688	17.7805
W-CNN-BiLSTM	0.9404	4.6816	3.2193	17.5674
W-CNN-LSTM-GRU	0.9464	4.5310	3.1840	16.5858
W-CNN-GRU-LSTM	0.9489	4.4583	2.7451	14.4150
W-CNN-BiLSTM-BiGRU	0.9859	2.5590	2.0056	10.0762
W-CNN-BiGRU-BiLSTM	0.9952	1.4935	1.2091	7.3782

## Data Availability

The raw data supporting the conclusions of this article will be made available by the authors upon request.
